# Elucidating pharmacodynamic interaction of silver nanoparticle - topical deliverable antibiotics

**DOI:** 10.1038/srep29982

**Published:** 2016-07-18

**Authors:** G. Thirumurugan, J. V. L. N. Seshagiri Rao, M. D. Dhanaraju

**Affiliations:** 1 School of Pharmaceutical Sciences and Technologies, Jawaharlal Nehru Technological University, Kakinada, AP, India; 2 Research Lab, GIET School of Pharmacy, Chaitanya Nagar, Rajahmundry, AP, India; 3 Department of Pharmaceutical analysis, AU College of Pharmaceutical Sciences, Andhra University, Visakhapatnam, AP, India

## Abstract

In order to exploit the potential benefits of antimicrobial combination therapy, we need a better understanding of the circumstances under which pharmacodynamic interactions expected. In this study, Pharmacodynamic interactions between silver nanoparticle (SNP) and topical antibiotics such as Cefazolin (CEF), Mupirocin (MUP), Gentamycin (GEN), Neomycin (NEO), Tetracycline (TET), Vancomycin (VAN) were investigated using the MIC test, Combination assay followed by Fractional Inhibitory concentration Index and Agar well diffusion method. SNP + MUP, SNP + NEO, SNP + VAN combinations showed Synergism (SN) and SNP + CEF, SNP + GEN, SNP + TET showed Partial synergism (PS) against *Staphylococcus aureus*. Four combinations (SNP + CEF, SNP + MUP, SNP + GEN, SNP + VAN) showed SN, SNP + TET showed PS and Indifferent effect (ID) were observed for SNP + NEO against *Pseudomonas aeruginosa*. SN was observed for SNP + CEF, SNP + GEN, SNP + NEO, SNP + TET and SNP + MUP showed ID, SNP + VAN showed PS against *Escherichia coli*. In addition, we elucidated the possible mechanism involved in the pharmacodynamic interaction between SNP-topical antibiotics by increased ROS level, membrane damage following protein release, K^+^ leakage and biofilm inhibition. Thus, our findings support that conjugation of the SNP with topical antibiotics have great potential in the topical formulation when treating complex resistant bacterial infections and where there is a need of more concentration to kill pathogenic bacteria.

Pharmacodynamic interactions are those where the effects of one drug are changed by the presence of another drug at its site of action. An interaction is said to occur when the effects of one drug are altered by the co-administration of another drug, herbal medicine, food, drink or other environmental chemical agents^1^. The net effect of the combination may manifest as an additive or enhanced effect of one or more drugs, antagonism of the effect of one or more drugs, or any other alteration in the effect of one or more drugs. Combinatorial antibiotic treatments can have diverse effects on bacterial survival. Antibiotics can be more effective as a combination treatment displaying either an additive effect (an effect equal to the sum of the treatments) or a synergistic effect (an effect greater than the sum of the treatments). The combination can also be antagonistic—that is, the effect of the combination treatment is less than the effect of the respective single-drug treatments^2^.

The use of Silver nanoparticles can be exploited in various fields, particularly medical and pharmaceutical due to their low toxicity to human cells, high thermal stability and low volatility^3^. This has resulted in a broad array of studies in which Silver nanoparticles have played a role as drug and as well as superior antimicrobial agent and even has shown to prevent HIV binding to host cells^4^. Researchers have investigated the synergistic effect of SNP when combined with other compounds: a combination of amoxicillin and SNP showed better antibacterial properties against Escherichia coli than when they were applied alone^5^. In addition, our previous study demonstrated that topically delivered biogenic SNPs formulation show superior wound healing efficacy when compared to standard silver sulphadiazine ointment currently available in the market^6^. Rather than using other delivery methods (oral or systematic), it is seen that silver is most commonly delivered through topical administration. Topical delivery of Silver metal has been used since ancient times for wound healing due to comparatively less toxic properties. Moreover, its surface properties provide the good conjugation ability with antimicrobial agents and easy impregnation on the cotton dressings. Incorporation of silver nanoparticle with topical antibiotics could provide broad coverage and be a better alternative against antibiotics resistant infection.

The present study aims to evaluate any pharmacodynamic interactions such as synergistic, additive, antagonistic effect of SNPs when conjugate with the commonly used topical deliverable antibiotics such as Cefazolin (CEF), Mupirocin (MUP), Gentamycin (GEN), Neomycin (NEO), Tetracycline (TET), Vancomycin (VAN) against major causes of wound, burn bacterial infections. In order to exploit the potential benefits of antimicrobial combination therapy, we need a better understanding of the circumstances under which pharmacodynamic interactions expected. In this study, we elucidated pharmacodynamic interactions by increased ROS level, membrane damage following protein release, K^+^ leakage and biofilm inhibition could allow for a more careful application of antibiotics that maintain the clinical capability and does not sacrifice the future usefulness of these drugs. To the best of the authors’ knowledge, this is the first report on the possible pharmacodynamic interaction of several topical antibiotics and SNPs.

## Results

### Preparation, characterization of the SNP

The current study utilizes the SNP synthesized biologically using potato plant pathogenic fungus *Phytophthora infestans*. The results of the SNP synthesis and characterization were described in our previous study^6^.

### MIC test

Antibacterial susceptibility pattern of SNP and various topical antibiotics against major causes of wound, burn bacterial infection such as *Staphylococcus aureus ATCC 25922, Pseudomonas aeruginosa ATCC 25619, Escherichia coli ATCC 10536* was evaluated by MIC test (Table 1). The MIC value of SNP for *S. aureus ATCC 25922*, *P. aeruginosa ATCC 25619, E. coli ATCC 10536* were 5, 2.5 and 2.5 μg/ml, respectively. Among the tested topical antibiotics; Cefazolin, Mupirocin, Neomycin and Vancomycin showed similar susceptibility (0.625 μg/ml) and Gentamycin, Tetracycline activity were also found to be similar level, 1.25 μg/ml against *S. aureus ATCC 25922*. Mupirocin, Vancomycin found to be more susceptible than other antibiotics tested and neomycin showed no activity against *P. aeruginosa ATCC 25619*. Cefazolin and Gentamycin showed similar activity, 0.3125 μg/ml followed by Tetracycline and Vancomycin, 0.625 μg/ml against *E. coli ATCC 10536* but this strain showed resistant against Mupirocin.

### Combination assay and FIC Index

Combination assay followed by FIC Index was measured to determine the pharmacodynamic interactions such as synergistic, partial synergistic and antagonistic or additive effects of SNP combined with topical antibiotics. Fractional Inhibitory concentration Index of SNPs and topical antibiotics against *S. aureus, P. aeruginosa, E. coli* were shown in the Table 2. Among the combinations tested against *S. aureus*; SNP + MUP, SNP + NEO, SNP + VAN showed synergistic (SN) effect and partially synergism (PS) were observed with SNP + CEF, SNP + GEN and SNP + TET. Interestingly, four combinations (SNP + CEF, SNP + MUP, SNP + GEN and SNP + VAN) showed synergistic efficacy and one SNP + TET showed PS but the FICI of SNP + NEO were found to be 1.75 μg/ml showed Indifferent (ID) efficacy against *P. aeruginosa*. Similarly, four combinations such as SNP + CEF, SNP + GEN, SNP + TET, SNP + NEO showed SN efficacy, one combination (SNP + VAN) showed PS and SNP + MUP showed an ID effect against *E. coli.*

### Agar well plate diffusion method

**Figure 1:**
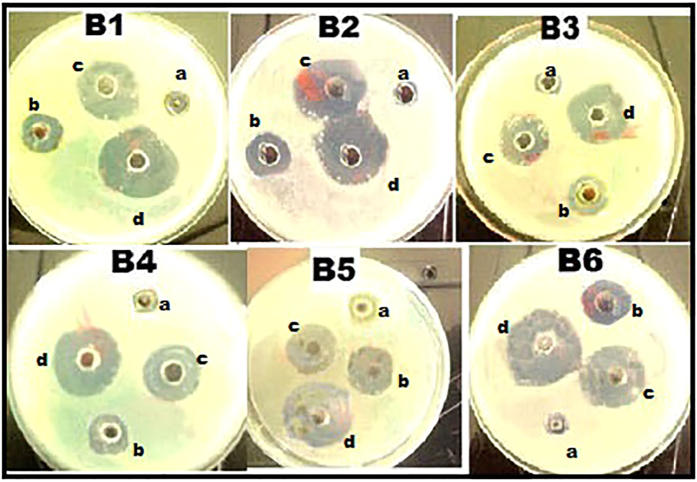
Petri plates showing the ZOI of SNPs, topical antibiotic and combinations against *S. aureus.* B1: Cefazolin; B2: Mupirocin; B3: Gentamycin; B4: Neomycin; B5: Tetracycline; B6: Vancomycin. a: Control; b: SNP; c: Topical antibiotic; d: SNP + Topical antibiotic.

**Figure 2:**
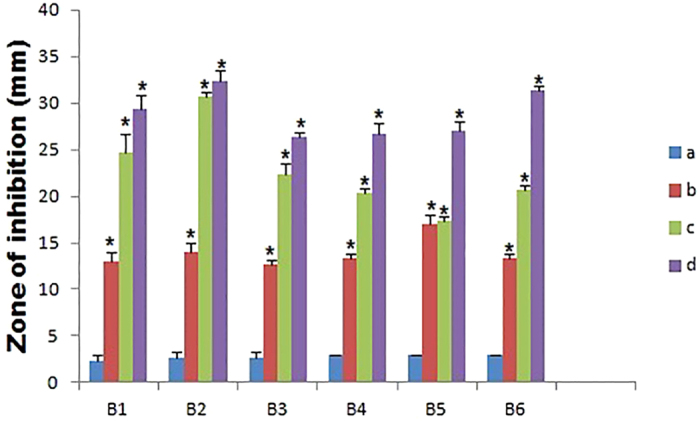
The Column chart showing the ZOI measurement of SNPs, topical antibiotic and combinations against *S. aureus*. B1: Cefazolin; B2: Mupirocin; B3: Gentamycin; B4: Neomycin; B5: Tetracycline; B6: Vancomycin. a: Control; b: SNP; c: Topical antibiotic; d: SNP + Topical antibiotic. Values are expressed as means (*n* = 3), and error bars represent standard deviations. Asterisks (*) indicate a statistical significant difference (*P* < 0.05) between the control and the treatments.

**Figure 3:**
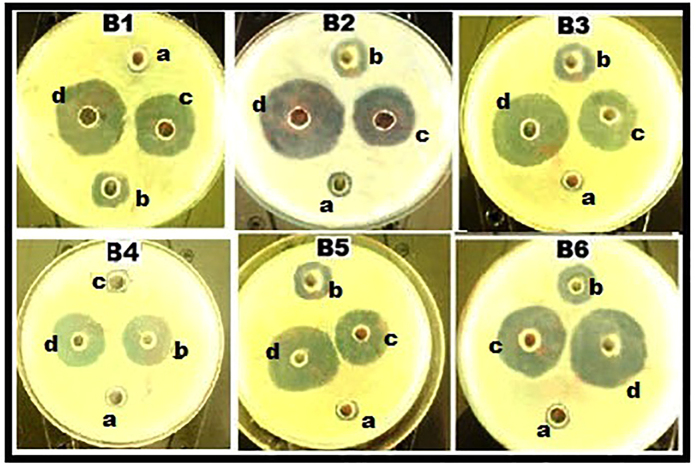
Petri plates showing the ZOI of SNPs, topical antibiotic and combinations against *P. aeruginosa*. B1: Cefazolin; B2: Mupirocin; B3: Gentamycin; B4: Neomycin; B5: Tetracycline; B6: Vancomycin. a: Control; b: SNP; c: Topical antibiotic; d: SNP + Topical antibiotic.

**Figure 4:**
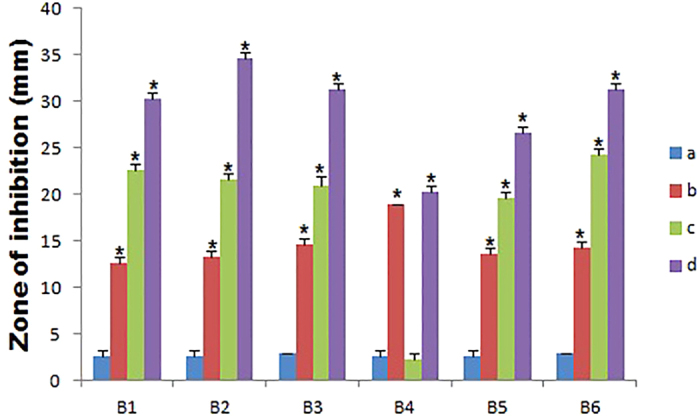
The Column chart showing the ZOI measurement of SNPs, topical antibiotic and combinations against *P. aeruginosa*. B1: Cefazolin; B2: Mupirocin; B3: Gentamycin; B4: Neomycin; B5: Tetracycline; B6: Vancomycin. a: Control; b: SNP; c: Topical antibiotic; d: SNP + Topical antibiotic. Values are expressed as means (*n* = 3), and error bars represent standard deviations. Asterisks (*) indicate a statistical significant difference (*P* < 0.05) between the control and the treatments.

**Figure 5:**
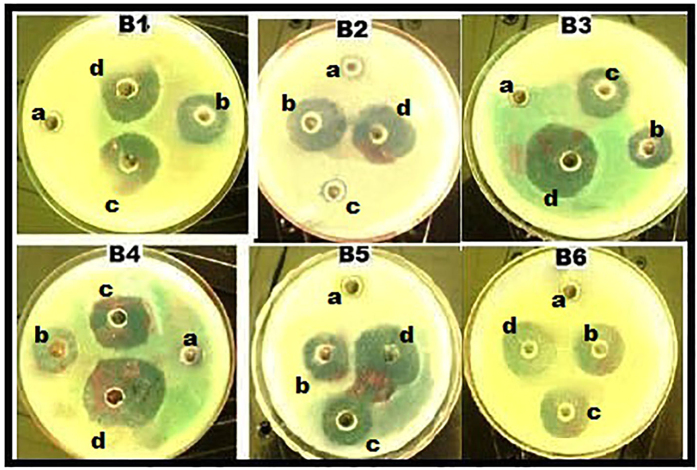
Petri plates showing the ZOI of SNPs, topical antibiotic and combinations against *E. coli*. B1: Cefazolin; B2: Mupirocin; B3: Gentamycin; B4: Neomycin; B5: Tetracycline; B6: Vancomycin. a: Control; b: SNP; c: Topical antibiotic; d: SNP + Topical antibiotic.

**Figure 6:**
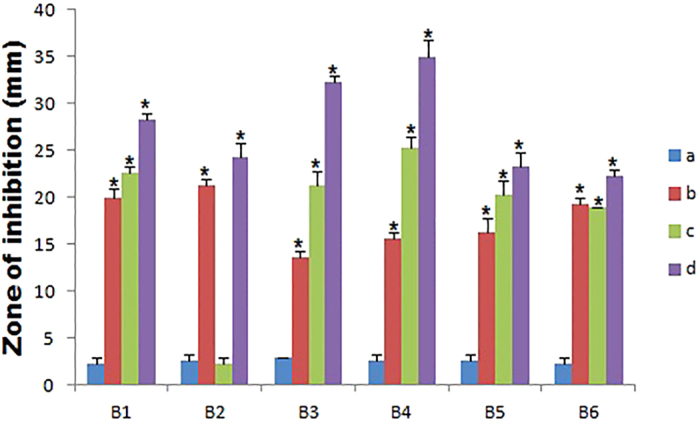
The Column chart showing the ZOI measurement of SNPs, topical antibiotic and combinations against *E. coli*. B1: Cefazolin; B2: Mupirocin; B3: Gentamycin; B4: Neomycin; B5: Tetracycline; B6: Vancomycin. a: Control; b: SNP; c: Topical antibiotic; d: SNP + Topical antibiotic. Values are expressed as means (*n* = 3), and error bars represent standard deviations. Asterisks (*) indicate a statistical significant difference (*P* < 0.05) between the control and the treatments.

Pharmacodynamic interactions of SNP combined with topical antibiotics were also evaluated by agar well plate method, Zone of inhibition (ZOI) of SNP or antibiotics alone and the combination were measured and compared with the control. Figure 1 shows the inhibitory effect of individual substance and combination against *S. aureus* and ZOI comparison was depicted in the Fig. 2. All individual and combinations were susceptible against *S. aureus* and highest ZOI 33, 32 and 31 mm were observed for the SNP + MUP, SNP + VAN and SNP + CEF respectively. In a case of individual or combinations treated against *P. aeruginosa*, Neomycin (concentration 2.5 μg/ml) alone showed no activity. However, SNP + NEO combination showed 21 mm ZOI and highest ZOI 35, 32 and 31 mm were observed for the SNP + MUP, SNP + GEN and SNP + VAN respectively. The inhibitory effect of individual substance and combination against *P. aeruginosa* were shown in the Fig. 3 and ZOI comparison was depicted in the Fig. 4. Similarly, strain *E. coli* also showed resistance against Mupirocin (concentration 0.625 μg/ml) but the combination SNP + MUP showed 26 mm ZOI and other combinations such as SNP + NEO, SNP + GEN showed ZOI about 36, 33 mm respectively. The results of the individual substance and their combinations against *E. coli* were depicted in the Figs 5 and 6.

### Comparative ROS formation assay

**Figure 7:**
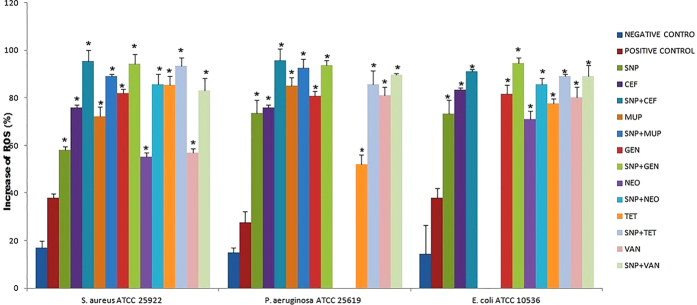
Comparative ROS formation assay. Values are expressed as means (*n* = 3), and error bars represent standard deviations. Asterisks (*) indicate a statistical significant difference (*P* < 0.05) between the negative control and the treatments.

Measurement of ROS generation was utilized to elucidate the pharmacodynamic interaction in this study since the elevated ROS formation leads to the imbalance redox system and can cause oxidative stress, the results of the comparative ROS formation assay were depicted in the Fig. 7. A significant (P > 0.05) increase in ROS formation was observed for the all three bacterial stain treated with the SNP and antibiotics alone as compared to the control bacterial strains. At the same time, the all combinations tested were shown 2–3 fold increased formation of ROS as compared to control for all bacterial strains.

### Comparative measurement of intracellular potassium release

**Figure 8:**
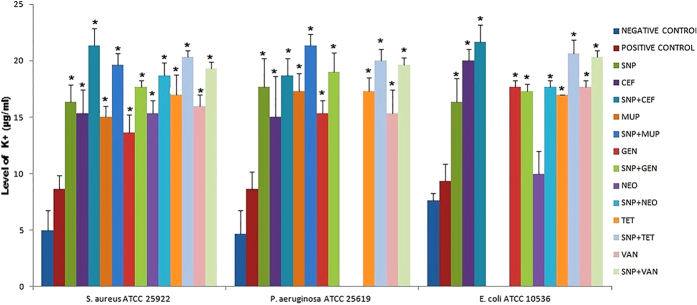
Comparative measurement of intracellular potassium release. Values are expressed as means (*n* = 3), and error bars represent standard deviations. Asterisks (*) indicate a statistical significant difference (*P* < 0.05) between the negative control and the treatments.

Measurement of intracellular K^+^ release was carried out to confirm bacterial membrane damage by individual substances and combination tested in this study. All individual substances, including SNP showed significant levels of intracellular K^+^ generation as compared to the negative and positive control against the strains *S. aureus* and *P. aeruginosa* (Fig. 8). But the Neomycin alone did not exhibit significant (p < 0.05) K^+^ levels as compared to the negative and positive control against the strain *E. coli.* Though Neomycin alone was not cause considerable K^+^ leakage, the still significant level was observed when combined with SNP (SNP + NEO) for the stain *E. coli.*

### Antibiofilm assay

**Figure 9:**
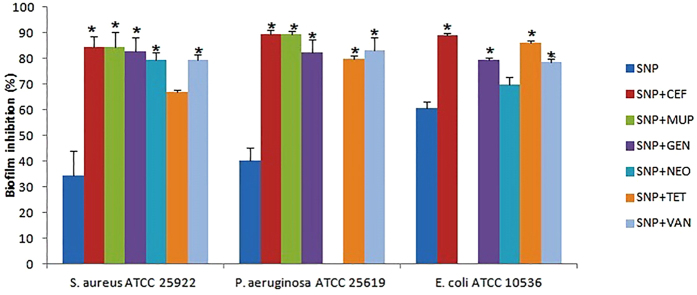
The inhibitory effect of SNP and combination with antibiotics on the biofilm formation. Values are expressed as means (*n* = 3), and error bars represent standard deviations. Asterisks (*) indicate a statistical significant difference (*P* < 0.05) between the SNP alone and the combinations.

Antibiofilm assay was performed for SNP alone and combined with topical deliverable antibiotics against three bacterial strains (Fig. 9). The results indicated that SNP mostly had an inhibitory effect on biofilm formation, an average of 60. 67 ± 2.52 against *E. coli* followed by 40.33 ± 5.033 for *P. aeruginosa* and 34.33 ± 9.71 against the strain *S. aureus*. The inhibitory effect of combinations with antibiotics on the biofilm formation was compared with the SNP alone, the results depicted that all combinations tested showed significant antibiofilm activity (P < 0.05) except the SNP combined with Neomycin (SNP + NEO) when compared to the SNP alone. Interestingly, the equal inhibitory effect was observed for SNP + CEF and SNP + MUP combinations against the strain *P. aeruginosa*.

### SDS-PAGE

**Figure 10:**
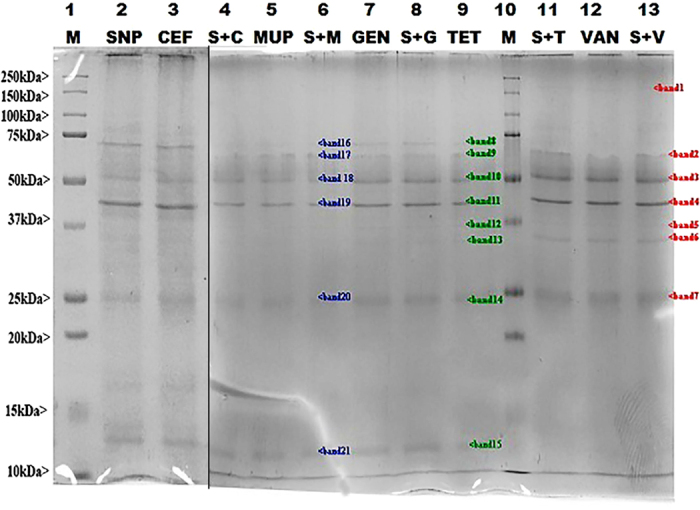
Membrane damage following intracellular protein caused by SNP, individual antibiotics and combination on *P. aeruginosa* cells visualized after SDS-PAGE and staining with coomassie. Lane 1, 10: Marker (M); Lane 2: SNP alone; Lane 3: CEF alone; Lane 4: SNP + CEF (S + C); Lane 5: MUP alone; Lane 6: SNP + MUP (S + M); Lane 7: GEN alone; Lane 8: SNP + GEN (S + G); Lane 9: TET alone; Lane 11: SNP + TET (S + T); Lane 12: VAN alone; Lane 13: SNP + VAN (S + V).

Membrane damage following intracellular protein caused by SNP, individual antibiotics and combination on *P. aeruginosa* cells were observed by SDS-PAGE assay. Lane 1, 10 was loaded with marker, 2 were loaded with SNP and 3, 5, 7, 9, 12 were loaded with individaul antibiotics, lane 4, 6, 8, 11, 13 were loaded with combinations. The results were visualized after SDS-PAGE and staining with coomassie as in Fig. 10. There were total of 22 bands visualized sizes of 12 kDa to 180 kDa after staining with coomassie. Among all bands, distinct bands were observed at 75 kDa, 50 kDa, 40 kDa intracellular protein caused by SNP, individual antibiotics and combination on *P. aeruginosa* cells.

### Microscopic comparison of live/dead cells

**Figure 11:**
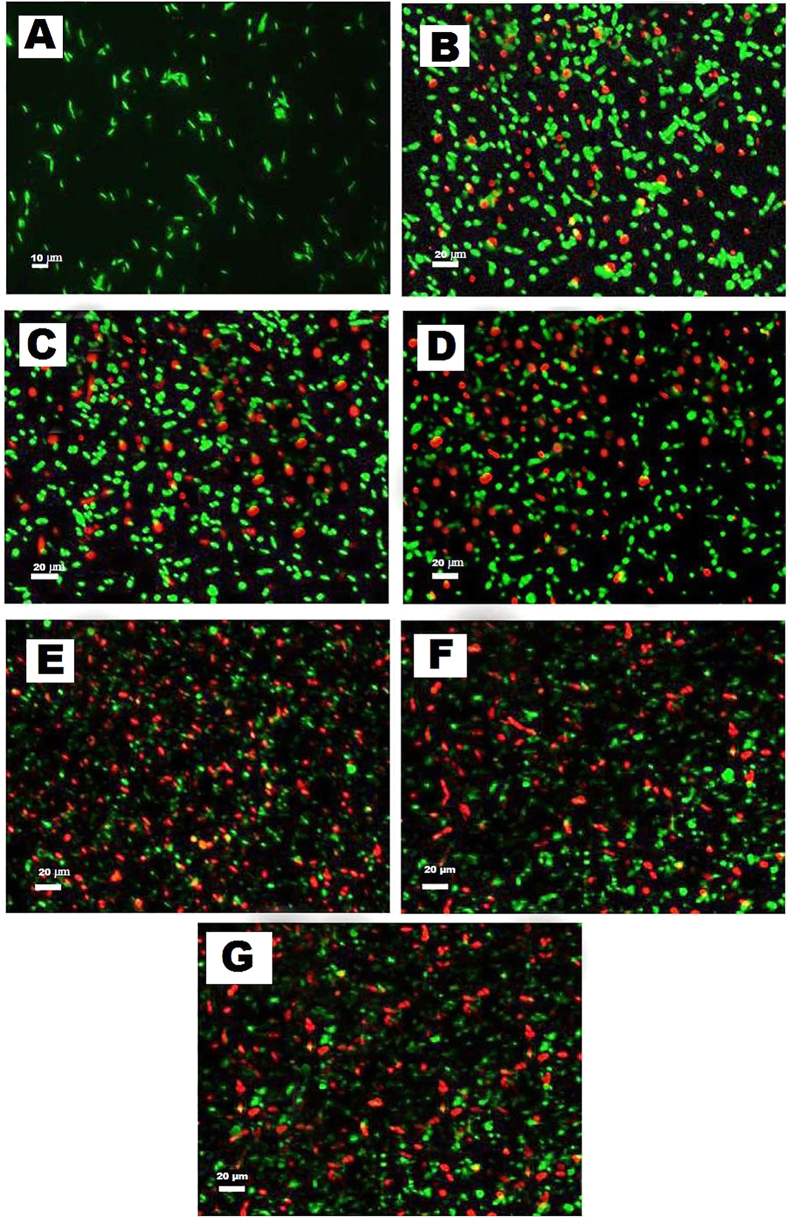
Results of LIVE/DEAD BacLight bacterial viability visualized in the fluorescence microscopy. *P. aeruginosa* cells treated with control (**A**) SNP alone (**B**) SNP + CEF (**C**) SNP + MUP (**D**) SNP + GEN (**E**) SNP + TET (**F**) and SNP + VAN. Cells with a damaged membrane showing red color fluorescence where as cells with an intact membrane showing green color fluorescence.

*P. aeruginosa* cells were treated with SNP or combination and control for 4 hrs and visualized in the fluorescence microscopy. *P. aeruginosa* cells with a damaged membrane showing red color fluorescence that considered to be dead, whereas cells with an intact membrane showing green color fluorescence. The results of LIVE/DEAD BacLight bacterial viability were shown in the Fig. 11. The cells treated with SNP alone (B) showed numerous red color as compared to the control (A), whereas the cells treated with combinations (C-G) showed an intense and higher amount of red color as compared to the control as well as SNP alone treated cells.

## Discussion

The discovery rate of new antibiotics is in decline, while antibiotic resistance in pathogens is rapidly increasing. Drug combinations offer potential strategies for controlling the evolution of drug resistance^2^. Despite their growing biomedical relevance, fundamental questions about drug interactions remain unanswered. In particular, little is known about the underlying mechanisms of most drug interactions. A better understanding of the circumstances under which pharmacodynamic interactions could allow for a more careful application of antibiotics that maintain the clinical capability and does not sacrifice the future usefulness of these drugs.

This study elucidates for the first time, pharmacodynamic interactions of SNP – topically deliverable antibiotics against major causes of wound, burn bacterial infections. To examine susceptibility patterns of the SNP, topical antibiotics alone and conjugation with SNP, MIC test was performed against *S. aureus, P. aeruginosa, E. coli*. Shrivastava *et al*.^7^ reported that the SNP were more susceptible against Gram-negative than Gram-positive bacteria^7^ and some strains of *E. coli* showed resistance against SNP^8^. In this study, SNP showed susceptibility against both Gram-negative and Gram-positive bacterial strains. All topical antibiotics tested were shown an inhibitory effect against *S. aureus*, Neomycin found to be not effective against *P. aeruginosa* and Mupiracin also not showed efficacy against *E. coli* strain. The previous studies stated that, antibacterial activities of few antibiotics were increased in the presence of SNP^9^, synergistic effects between polymyxin B and silver nanoparticles for gram-negative bacteria^10^ and Cephalexin conjugated Silver nanoparticles against *E. coli*^11^.

The current study evaluated the entire pharmacodynamic interaction between SNP and topical antibiotics, results of combined assay followed by FIC Index and agar well diffusion assay showed that various degrees of pharmacodynamic interactions such as SN, PS and ID against a common cause of infections in wound, burns. Interestingly, antagonistic (AN) effect was not observed among the combinations against all tested bacterial strains in this study. However, no report was found in the literature showing the antagonistic effect of SNP with antiinfectives. Our study results suggested that the SNP could be conjugated with an anti-infective agents having less or no efficacy, an agents need more concentration to kill pathogenic bacteria and to target both gram-positive/negative resistant bacterial strains causing complex infections.

In order to elucidate the pharmacodynamic interactions observed in this study, we conducted the comparative ROS formation assay, Comparative measurement of intracellular potassium release, anti-biofilm assay, SDS-PAGE and Microscopic comparison of live/dead cells. Kohanski M. A. *et al*.^12^ revealed that the mechanism of ROS formation induced by bactericidal antibiotics is the end product of an oxidative damage cellular death pathway and additionally, In-sok Hwang. *et al*.^13^ reported that two different classes of bactericidal antibiotics, ampicillin and kanamycin, induced ROS formation^13^. Our findings consistent with this, there was a statistically significant (p < 0.05) increase of ROS in the bacterial strains treated with SNP alone and combinations, suggested that ROS generated membrane damage could be a major cause for pharmacodynamic interaction seen. Bortner and Cidlowski^14^ report that, when the concentration of intracellular potassium (K+) is normal, the cell death process is repressed by the suppression of the caspase cascade and inhibition of the apoptotic nucleases activity and Tiwari *et al*.^15^ correlated that the leakage of intracellular potassium due to membrane damage. Our findings revealed that SNP and combinations caused significant level potassium leakage due to membrane damage as compared to the control treated against *S. aureus, P. aeruginosa, E. coli*.

Biofilms are microbial communities consisting of cells attached to biotic or abiotic surfaces, embedded in an exopolymeric matrix. These structures are well known for their remarkable resistance to diverse chemical, physical, and biological antimicrobial agents and are one of the main causes of persistent infections^16^. Chan *et al*. have shown that alginate, a polysaccharide of *P. aeruginosa* biolfim matrix has an important role in *P. aeruginosa* resistance^17^. In this study, significant biofilm inhibition was observed for all combinations tested, however SNP also were shown a good degree of antibiofilm activity, suggested that the SNP could be conjugated when resistance of bacteria due to biofilm formation and SNP conjugation provide more potential to achieve greater biofim inhibition at lower antibiotic concentrations. Membrane damage following intracellular protein bands was observed by SDS-PAGE, however the protein bands obtained must be subjected to protein peptide mass fingerprinting analyses would provide specific clues and damage of the membrane caused by SNP alone, the combinations were visualized in the fluorescence microscopy. These findings revealed that SNP, conjugated antibiotics caused membrane damage following intracellular proteins and bacterial cell death visualized in fluorescence microscopy.

## Conclusion

This study presented pharmacodynamic interaction between SNP and topical antibiotics such as synergism, partial synergism and indifferent effect against the major cause of wound infections. Moreover, we elucidated the possible mechanism involved in the pharmacodynamic interaction caused by increased ROS level, membrane damage following protein release, K+ leakage and biofilm inhibition. Thus, our findings support that conjugation of the SNP with topical antibiotics have great potential in the topical formulation when treating complex resistance, bacterial infections and where there is a need of more concentration to kill pathogenic bacteria.

## Materials and Method

### Agents, bacterial strains and culture conditions

All antibiotics used in this study (Cefazolin, Mupirocin, Gentamycin, Neomycin, Tetracycline, Vancomycin) were purchased from Sigma Chem. Co., USA. *Staphylococcus aureus ATCC 25922, Pseudomonas aeruginosa ATCC 25619, Escherichia coli ATCC 10536* were obtained from the American Type Culture Collection. LB medium purchased from Oxoid, UK was used for the bacterial cell growth at 37 °C and growth was monitored by measuring the optical density at 620 nm.

### Preparation, characterization of SNP

The current study utilizes the SNP synthesized biologically using potato plant pathogenic fungus *Phytophthora infestans*. Method of synthesis and characterization of the SNP is described in our previous study^6^.

### MIC test

Susceptibility tests with SNPs, Cefazolin, Mupirocin, Gentamycin, Neomycin, Tetracycline, Vancomycin were carried out in 96-well microtitre plates using a standard twofold broth micro dilution method in Mueller Hinton (MH) broth (HiMedia), referring Clinical and Laboratory Standards Institute guidelines^18^. Briefly, bacterial cells were grown to midexponential growth phase in MH medium. 100 ml of the bacterial cells was then added in the wells of a 96-well microtitre plate at a density of 1 × 10^6^ ml^−1^; 10 ml each of the serially diluted solutions of the compounds was then added to the bacterial cells. The MIC was defined as the lowest concentration of the agent/drug inhibiting visible growth after overnight incubation at 37 °C.

### Combination assay

The MICs of each antibiotic substance alone or in combination were determined by a broth microdilution method in accordance with CLSI standards using cation-adjusted MH broth, modified for a broth microdilution checkerboard procedure^18^. For the double treatment, a 2D checkerboard with twofold dilutions of each drug was used to test the different combinations as follows. A checkerboard with twofold dilutions of SNPs and antibiotic (Cefazolin, Mupirocin, Gentamycin, Neomycin, Tetracycline, Vancomycin) were set up as described above for the combined treatment. Growth control wells containing the medium were included in each plate.

### Fractional Inhibitory concentration (FIC) and FIC Index

To determine the effectiveness of test substances for synergistic, antagonistic or additive effects, FIC index was measured, using the following formulas^19^. FIC of drug A = MIC drug A in combination/MIC drug A alone, and FIC of drug B = MIC drug B in combination/MIC drug B alone. The FIC index (FICI), calculated as the sum of each FIC, was interpreted as follows: synergistic (≤0.5), partially synergistic (>0.5 to 1), additive (Equal to 1), indifferent (>1 to <2) or antagonistic (≥2) on the basis of FIC.

### Agar well plate diffusion method

Pharmacodynamic interaction was confirmed by agar well plate diffusion method^20^ and according to the guidelines of the National Committee for Clinical Laboratory Standards^18^. Bacterial cell 1 × 10^6^ ml^−1^ density were treated with each substance or combination so that the final concentration was the MIC or FIC, respectively. Pharmacodynamic interaction, such as synergism, additive, indifferent and antagonistic effect were defined as comparing the zone of inhibition surroundings of well containing topical antibiotic, antibiotic + SNPs and control.

### Comparative intracellular ROS formation assay

Bacterial cells at a density of 1 × 10^6^ ml^−1^ were treated with each substance or combination so that the final concentration was the MIC or FIC, respectively. Samples were incubated for 1 h at 37 °C. The negative and positive controls were maintained with untreated and 70% ethanol (50 μL/mL) treated cells, respectively. To detect the ROS formation, we used the fluorescent reporter dye 2,7-dichlorodihydrofluorescein diacetate (Sigma, Bengaluru, India) at a concentration of 5 mM and used a spectrofluorometer (Shimadzu) to measure excitation at a wavelength of 490 nm and emission at 515 nm. The level of reactive oxygen species increase was calculated using the equation: 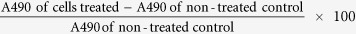


Experiments were performed in triplicate and means ± SD were calculated^12^.

### Comparative measurement of intracellular potassium release

The assessment of the potassium ions present in medium was carried out using a luminescence spectrometer (Perkin-Elmer) at an excitation wavelength of 346 nm and an emission wavelength of 505 nm. The cells in exponential phase were obtained, the potassium sensitive probe (PBFI, Sigma Aldrich) was added and treated with a substance or combination followed by incubation at 37 °C for 4 h. The negative and positive controls were maintained with untreated and 70% ethanol (50 μL/mL) treated cells, respectively. The cells were measured following separation of cellular debris by centrifugation at 4000 rpm.

### Antibiofilm assay

190 μl bacterial cells at a density of 1 × 10^6^ ml^−1^ were added in the 96- well polystyrene sterile flat bottom tissue culture plates and incubated for 18 hrs. 10 μl SNP alone or combination so that the final concentration was the MIC or FIC, respectively, and incubated at 37 °C for 6 hrs. The tissue culture plates were washed with sterile phosphate-buffered saline for the removal of floating bacteria and sessile adherent bacteria were fixed using 2% sodium acetate, 0.1% crystal violet stain. Tissue culture plates were washed with deionized water and 95% ethanol was added to the wells after drying. The absorbance of stained adherent bacteria was recorded at 595 nm ELISA reader (Molecular Devices). The % of antibiofilm activity was calculated using the equation: 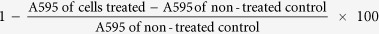 and means ± SD were determined.

### Intracellular protein measurement

The pharmacodynamic effect of SNP and combination on *P. aeruginosa* cells were observed by SDS-PAGE assay according to the method^21^. Gels were run on a Mini Protean III vertical electrophoresis system (BioRad) at 100 V until the tracking dye of the sample buffer reaches to the other end. The gels were stained with 0.1% coomassie blue R-250 in 10% acetic acid and 40% methanol, destained in 10% acetic acid at 40% methanol.

### Microscopic comparison of live/dead cells

The pharmacodynamic effect of the SNP or combination and control was also observed microscopically using Live/dead assay kit (Thermo Fisher Scientific). *P. aeruginosa* cells were treated with SNP or combination and control for 4 h. Later, the cells were washed, resuspended in sterile phosphate-buffered saline and stained with LIVE/DEAD BacLight bacterial viability kit as per manufacturer’s instructions.

### Statistical analysis

Three independent experiments were carried out and the results are represented as mean ± SD. The Student’s T test was applied to calculate the statistical significance of the experimental data.

## Additional Information

**How to cite this article**: Thirumurugan, G. *et al*. Elucidating pharmacodynamic interaction of silver nanoparticle - topical deliverable antibiotics. *Sci. Rep.* **6**, 29982; doi: 10.1038/srep29982 (2016).

## Figures and Tables

**Table 1:** Antibacterial susceptibility (MIC) patterns of SNP and various topical antibiotics against major causes of wound, burn bacterial infections.

Bacterial strain	SNP	Cefazolin	Mupirocin	Gentamycin	Neomycin	Tetracycline	Vancomycin
	MIC (μg/ml)						
*S. aureus ATCC 25922*	5 μg/ml	0.625 μg/ml	0.625 μg/ml	1.25 μg/ml	0.625 μg/ml	1.25 μg/ml	0.625 μg/ml
*P. aeruginosa ATCC 25619*	2.5 μg/ml	0.625 μg/ml	0.3125 μg/ml	0.625 μg/ml	£	1.25 μg/ml	0.3125 μg/ml
*E. coli ATCC 10536*	2.5 μg/ml	0.3125 μg/ml	£	0.3125 μg/ml	2.5 μg/ml	0.625 μg/ml	0.625 μg/ml

£- no activity; SNP- silver nanoparticle; MIC- Minimum Inhibitory Concentration.

**Table 2:** Fractional Inhibitory concentration Index of SNPs and topical antibiotics.

SNPs + Topical Antibiotics	FICI		
	*S. aureus ATCC 25922*(Inference)	*P. aeruginosa ATCC 25619*(Inference)	*E. coli ATCC 10536*(Inference)
SNP + CEF	0.6 μg/ml (PS)	0.3 μg/ml (SN)	0.5 μg/ml (SN)
SNP + MUP	0.3 μg/ml (SN)	0.1 μg/ml (SN)	1.75 μg/ml (ID)
SNP + GEN	0.6 μg/ml (PS)	0.4 μg/ml (SN)	0.3 μg/ml (SN)
SNP + NEO	0.3 μg/ml (SN)	1.75 μg/ml (ID)	0.5 μg/ml (SN)
SNP + TET	0.6 μg/ml (PS)	0.8 μg/ml (PS)	0.3 μg/ml (SN)
SNP + VAN	0.3 μg/ml (SN)	0.3 μg/ml (SN)	0.6 μg/ml (PS)

SN- Synergistic; PS- Partially Synergistic; AD- Additive; ID- Indifferent; AN- Antagonistic; Synergistic (≤0.5), Partially synergistic (>0.5 to 1), Additive (Equal to 1), Indifferent (>1 to <2) or Antagonistic (≥2) on the basis of FIC.
